# In Vivo Digestion of Egg Products Enriched with DHA: Effect of the Food Matrix on DHA Bioavailability

**DOI:** 10.3390/foods10010006

**Published:** 2020-12-22

**Authors:** Carlos Pineda-Vadillo, Françoise Nau, Catherine Guérin-Dubiard, Claire Bourlieu, Francesco Capozzi, Alessandra Bordoni, Didier Dupont

**Affiliations:** 1 STLO, INRAE, Institut Agro, 35042 Rennes, France; carlos.pinedavadillo@mixscience.eu (C.P.-V.); catherine.guerin@agrocampus-ouest.fr (C.G.-D.); didier.dupont@inrae.fr (D.D.); 2 IATE, Univ Montpellier, CIRAD, INRAE, Institut Agro, 34060 Montpellier, France; claire.bourlieu-lacanal@inrae.fr; 3 Interdepartmental Centre for Industrial Agri-Food Research, University of Bologna, 47521 Cesena, Italy; francesco.capozzi@unibo.it (F.C.); alessandra.bordoni@unibo.it (A.B.); 4 Department of Agri-Food Sciences and Technologies, University of Bologna, 47521 Cesena, Italy

**Keywords:** DHA, bioavailability, in vivo digestion, food matrix, egg

## Abstract

The aim of the present study was to determine to what extent the food matrix could affect the release of docosahexaenoic acid (DHA) during digestion and its incorporation into systemic circulation. In this aim, three DHA-enriched egg products having the same composition but different structure were developed: omelet, hard-boiled egg, and mousse. Then, nine pigs fitted with T-shape cannulas at duodenal level and a jugular venous catheter were fed with the DHA-enriched egg products, and duodenal effluents and plasma were collected throughout the postprandial period. Results highlighted an undeniable effect of the food matrix on digestion parameters and DHA bioavailability. The transit of DHA and protein through the duodenum was faster after the ingestion of the mousse than after the ingestion of the omelet and hard-boiled egg. While most of the DHA and protein ingested under the form of mousse had already passed through the duodenum 4.5 h after its ingestion, significantly higher quantities were still present in the case of the omelet and hard-boiled egg. In terms of bioavailability, the omelet was the most efficient vector for delivering DHA into systemic circulation. It supplied 56% and 120% more DHA than the hard-boiled egg and the mousse, respectively.

## 1. Introduction

DHA (docosahexaenoic acid) is the longest and most unsaturated fatty acid of the natural omega-3 polyunsaturated fatty acid family (n-3 PUFA). Commonly designated as C22:6 n-3, DHA is a critical component of all cell membranes, playing a crucial role in maintaining membrane integrity and fluidity [1]. DHA is especially involved in visual and neural function, as well as in neurotransmitter metabolism [2,3].

Apart from the benefits derived from its structural role, DHA is related to a myriad of other health benefits. The intake of DHA and n-3 PUFA has been associated with the prevention of cardiovascular disease and its risk factors. In particular, n-3 PUFA may modulate adipose tissue function [4,5], increase HDL-C while lowering total cholesterol [6,7,8], decrease high blood pressure [9,10,11], lower blood triglycerides [12,13,14,15], modulate insulin resistance [16], counteract oxidative stress at adequate dose [17], and protect against endothelial dysfunction [18,19]. Additionally, DHA and n-3 PUFA may also alleviate pain and inflammation related to arthritis [20,21], decrease cancer cell survival [21,22,23,24,25], reduce asthma symptoms [26,27], and reduce the risk of Alzheimer’s disease, dementia, and cognitive dysfunction [28,29,30,31,32].

According to several studies, the consumption of DHA in many Western countries would be around 100 mg per day [33,34,35,36], i.e., significantly lower than the optimal recommendations (250 mg to 500 mg of combined EPA and DHA per day for healthy adults [37,38]). Although α-linolenic acid (C18:3 n-3), the precursor molecule of DHA, can be ingested through vegetables and vegetable oils in substantial quantities, its endogenous conversion rate into DHA is very low, dependent among other factors on the concentration of n-6 FA in the diet [39] and can be lowered in certain physiological states (ca. 5%) [40]. Therefore, consuming DHA directly from foods is the only practical way to increase its intake. Consumption of fish and seafood, the main sources of DHA in the human diet, is low in Western countries, making DHA supplements and fortified foods a good alternative to increase or complete the recommended intake.

DHA enriched-eggs, conventional eggs fortified with DHA through flax or marine byproducts fed to laying hens, are a very interesting choice to increase DHA intake. Eggs are natural, inexpensive, can be eaten on a daily basis and are accepted worldwide by all age groups. From a nutritional point of view, DHA-enriched eggs may contain up to five times more omega-3 than conventional eggs and 39% less arachidonic acid (C20:4 n-6), an inflammatory n-6 PUFA [41]. As an example, whole egg prepared in the present study, using the commercial DHA-enriched egg yolk powder (Section 2.2) contained around 370 mg DHA (plus 26 mg EPA) per 100 g of product, thus making a significant contribution toward the recommended target intake mentioned above for long chain PUFA. In addition, DHA is mainly under the form of phospholipids in eggs, which leads to higher bioavailability than triglycerides, the main source of n-3 PUFA in fish oil and its derived products [42,43,44,45].

The real effectiveness of food bioactives depends mainly on four steps: their release in the gastrointestinal tract, their intestinal absorption, their metabolism, and finally their health effect. In this context, the food matrix may either enhance or prevent the release and the solubilization of DHA during digestion, and hence its bioavailability and effectiveness. Thus, the issue of the interactions between the food matrix and DHA is of crucial importance during the development of potential effective enriched-foods, but at the end very little addressed.

An in vitro study demonstrated that the release of both EPA and DHA during digestion was different depending on how these PUFAs were ingested: alone as a microencapsulated tuna oil powder or included into three different food matrices (orange juice, yogurt, or cereal bar) [46]. In a randomized cross-over study that included 12 healthy male participants, Schram et al. [47] observed that when a fish oil was ingested as a supplement or incorporated into different food matrices (a fitness bar, a yogurt drink, bread and butter), the bioavailability of n-3 PUFA was influenced by the matrix of the food product.

However, in vivo studies on the bioavailability of DHA depending on the food in which this PUFA is included are quite rare, and, to the best of our knowledge, no one compared foods with same composition but different structures. Then, the issue of the impact of the food structure on the nutrient bioaccessibility and bioavailability is a specific one, which we decided to explore in the present study.

In that aim, three DHA-enriched egg products having the same composition but different structures were developed: omelet, hard-boiled egg, and mousse. Then, nine pigs fitted with T-shape cannula at duodenal level and a jugular venous catheter were fed with the DHA-enriched egg products. Duodenal effluents and plasma were collected throughout the postprandial period (over 7.5 h for duodenal effluents, up to 48 h for plasma). Duodenal pH, protein concentration, proteolysis, and DHA concentration, as well as plasma DHA concentration were followed-up over the digestion. Duodenal protein concentration and proteolysis were regarded as indicators for gastric emptying and food disintegration, respectively.

## 2. Materials and Methods

### 2.1. Chemicals

Unless otherwise stated, chemicals were purchased from Sigma (St Louis, MO, USA). Ultrapure water was purified using a Milli-Q system (Millipore, Molsheim, France). Identification and quantification of fatty acids was performed using the FAME kit Mixture ME 100, 10 mg of Larodan (Solna, Sweden).

### 2.2. DHA-Enriched Test Meals

The DHA used to fortify the matrices of the study was supplied by Applications Santé des Lipides-ASL (Vichy, France). The product, registered as OVO-DHA^®^, consisted of a DHA-enriched egg yolk powder obtained after spray drying of pasteurized DHA-enriched egg yolks. The accumulation of DHA in the yolks was naturally obtained after the feeding of hens with a selection of foods inherently rich in PUFAs. Almost all the DHA (98%) was included in phospholipids, in *sn*-2 position. In order to avoid differences among the products, all the OVO-DHA^®^ used in the study came from a single batch. OVO-DHA^®^ fatty acid composition is shown in Table 1.

Three egg matrices having a similar composition but different structure, namely, omelet, hard-boiled egg, and mousse, were prepared maintaining the natural white:yolk ratio found in eggs, i.e., 68:32 *w/w* [48]. Omelets were prepared by filling 45 mm diameter polyvinylidene chloride (PVDC) casings (Krehalon Ind., Deventer, The Netherlands) with a homogeneous mixture of 160 g of previously rehydrated egg yolk powder (72 g OVO-DHA^®^ plus 88 g water according to the supplier recommendation) and 340 g of pasteurized liquid egg white from Liot^®^ (Pleumartin, France). After their tight closing, they were cooked during 30 min at 86 °C in a water bath in order to obtain an egg gel firm enough to be easily cut into pieces of controlled size, and then cooled down to room temperature before storage at 4 °C until use. Hard-boiled eggs were prepared similarly, with the solely exception that the egg yolk and the egg white were cooked separately in independent PVDC casings, before combining pieces of gelled egg white and gelled yolk in the same white: yolk ratio as mentioned above for omelet. Omelets and hard-boiled eggs were cooked 24 h before the pigs feeding. Just before feeding the PVDC casings were removed, and the matrices sliced into 1–2 cm thick pieces. The mousse was obtained by beating the pasteurized liquid egg white to a stiff peak stage with a Hobart N50 stand mixer at maximum speed during 3 min. Then the rehydrated egg yolk was added and mixed gently until a homogeneous mousse was obtained. Mousse, in order to keep its foamy structure by the time of ingestion, was prepared 1 h before pigs’ feeding.

### 2.3. Animals and Animal Housing

All experimental methods were in accordance with the guidelines formulated by the European Community for the use of experimental animals (L358-86/609/EEC). The study (Project N° 2015090309151893) received prior approval (on 18 January 2016) by the Local Committee for Ethics in Animal Experimentation.

The study involved nine adult Large White × Landrace × Piétrain female pigs (34.4 ± 2.0 kg). Three weeks before the experimentation, pigs were surgically fitted with a T-shaped cannula (silicone rubber) in the duodenum (10 cm downstream from the pylorus) and a catheter (polyvinyl chloride; 1.1 mm internal diameter, 1.9 mm outer diameter) in the jugular vein (around 10 cm inside the blood vessel). Pre-anesthesia of the animals was achieved by an intramuscular injection of ketamine at 15 mg/kg (Imalgene 1000^®^). Surgical procedures were performed under intubation anesthesia with an oxygen/isoflurane gas mixture. During the intervention, analgesia of the animals was achieved by the intravenous infusion at ear level of Fentanyl Renaudin^®^ (10 mL at 50 µg/mL). Animals received an intramuscular dose of amoxicillin the day of the operation, and then two more after 48 and 96 h (Duphamox Long Acting^®^, 150 mg/mL; at a dose of 1 mL/10 kg body weight). In case of post-surgical discomfort, analgesia continued during 2 days by a subcutaneous injection of morphine hydrochloride every 2 h (0.1 to 0.5 mL). Furthermore, in case of post-surgical discomfort, pigs were given Spasfon^®^ for abdominal pain (1 ampoule of 4 mL twice a day) and paracetamol mixed with the food twice a day (Pracétam^®^ 10%) at a dose of 30 mg/kg body weight.

Pigs were housed in individual slatted pens (1 m^2^) within a ventilated room with controlled temperature (21 °C). These conditions allowed the pigs to see, feel, and hear each other. Between the sampling days, pigs were fed twice daily with 800 g/d of a concentrate feed containing wheat (32.2%), corn (15%), barley (25%), wheat bran (5%), rapeseed (7%), soy (11.5%), vegetal fat (1%), calcium carbonate (1.5%), di-calcium phosphate (0.1%), NaCl (0.45%), lysine (0.53%) methionine (0.04%), L-threonine (0.1%), tryptophan 20% (0.07%), and phytase (0.01%). The days before sampling, pigs were fed only once daily with 400 g/d of the concentrate feed. Pigs had free access to water.

### 2.4. Experimental and Sampling Procedures

The experimental protocol included two periods: the first one for the duodenum effluent sampling, and the second one for blood sampling. In each one, the three egg matrices were randomly tested on each pig. Within a period (2 weeks), the days of sampling were separated by at least 48 h. Test meals (500 g of freshly prepared matrices) were entirely consumed within 2–5 min. Pigs had no access to water from 1 h before to 7.5 h after the meal delivery.

Duodenum effluent samplings were collected 15 min before and 2 min, 20 min, 50 min, 1.5 h, 3 h, 4.5 h, 6 h, and 7.5 h after the beginning of meal ingestion. The sampling was stopped when at least 20 mL of effluents were collected (this was always completed in less than 5 min). Collected effluents were vortexed and divided into two aliquots. The first one (around 15 mL) was used for pH measurement, and de visu morphologic characterization which consisted in spreading it into a Petri dish, recording any visual color change in comparison with the basal situation, as well as the appearance of food matrix pieces as the case may be. The biggest food pieces were measured manually using a graduated ruler. The second one (4 mL) was used for chemical analyses after a prior preparation as follows. Effluent samples were homogenized for 1 min at 13,000 rpm with an IKA T-25 Ultraturrax digital disperser (IKA, Staufen, Germany) and transferred into tubes containing 80 µL of a lipase inhibitor mixture. The mixture, prepared according to Hernell et al. [49], consisted in a methanolic solution of 50 mM di-isopropylfluorophosphate, 50 mM di-ethyl (p-nitrophenyl) phosphate, 50 mM acetophenone, and 250 mM phenylboronic acid. In addition to the lipase inhibition cocktail, an aqueous solution of 8% NaN_3_ (*w/w*) and 10% chloramphenicol (*w/v*) (5 µL per milliliter of effluent) was added to prevent bacteria growth. In order to avoid DHA and lipid oxidation, a methanolic solution of butylated hydroxytoluene (BHT) was added to a final concentration of 100 µM BHT. Finally, in order to stop proteolysis, pepstatin A and pefabloc were added to final concentrations of 10 µM and 5 mM, respectively. After vortexing, four aliquots of around 1 mL each were stored at −20 °C until analysis.

Blood sampling was performed 15 min before and 1 h, 2 h, 3 h, 4 h, 6 h, 8 h, 10 h, 24 h, and 48 h after the beginning of meal ingestion. Blood samples (3 mL) were collected in Venosafe^®^ VF-053SHL tubes containing 45 USP U lithium-heparin (Terumo, Shibuya, Tokyo, Japan) and immediately placed on ice. Within the same day, all tubes were centrifuged at 3000× *g* for 10 min at 4 °C. Supernatant (plasma) was collected, added BHT (10 µL of a 10.1 mM methanolic solution per mL of plasma) and stored at −80 °C until analysis.

### 2.5. Chemical Analyses

#### 2.5.1. Lipid Extraction from Food Matrices

In order to determine that no DHA was lost during the preparation of the matrices, especially in the cooked ones, the recovery of DHA in the ready-to-eat matrices was calculated. Three individual portions of each egg matrix were prepared and quantified individually (*n* = 3).

After freeze-drying of the matrices, lipids were extracted according to the Folch method [50]. Around 2 g of each freeze-dried matrix were precisely weighed, added with 6 mL water, and placed for 15 min in an ultrasonic bath. Then, after addition of 45 mL of Folch solvent (chloroform:methanol 2:1 *v/v*) and a known quantity of an internal standard (1,2-ditridecanoyl-*sn*-glycero-3-phosphatidylcholine, C13:0), the mixture was homogenized for 2 min at 10,000 rpm with an UT25 basic Ultraturrax^®^ disperser (IKA, Staufen, Germany). The whole mixture was then transferred into a 250 mL separation funnel; a final rinsing with 15 mL of Folch solvent was performed to ensure a complete transfer. After shaking vigorously, the mixture was left for 15 min in contact with the solvent and then added with 9 mL of a 0.73% NaCl solution (*w/v*) in order to reach the final Folch conditions and help deproteinization. After shaking and a 30 min waiting time, the lower phase was filtered (Whatman^®^ ashless grade 42 filter, Fisher Scientific SAS, Illkirch, France) and collected in a small amount (1–2 g) of anhydrous Na_2_SO_4_. Then, a mixture containing 60 mL of Folch solvent and 15 mL of 0.58% NaCl solution (*w/v*) was added to the separation funnel. After a waiting time of 30 min for dephasing, the lower phase was collected as described above. This step was performed twice. The lipid fraction was recovered after complete evaporation of the solvent (constant weight) in a Heidolph Hei-VAP Platinum 2 Rotary Evaporator (Schwabach, Germany) (55 °C, 474 mbar, 200 rpm), and a final dehydration for 10 min at 102.5 °C. Lipid quantity was then determined gravimetrically. Lipid fractions were stored at −20 °C until gas chromatography analysis.

#### 2.5.2. DHA Quantification in Food Matrices, Effluents, and Plasma

Total DHA in the effluents and plasma samples was analyzed by gas chromatography coupled to a flame ionization detector (GC–FID), after direct trans-methylation in the presence of internal standard (C13:0) and without prior extraction. Total DHA in the food matrices was quantified by the same technique except trans-methylation was performed in the fat extracts obtained by Folch extraction and dilution in chloroform as described in Section 2.5.1.

Trans-methylation was carried out according to Lopez et al. [51]. In brief, 0.5 mL of chloroform, 200 μL of sample (effluent, plasma or fat extract), 20 µL of C13:0 standard (0.5 mg/mL in chloroform, only for effluents and plasma), and 1 mL of 0.5 M sodium methoxide were added in a screw-capped tube. After a flush of nitrogen, caps were closed, and tubes were vortexed and placed in a Stuart SBH200D dry bath (Bibby scientific France, Villepinte, France) at 50 °C for 10 min. Tubes were cooled to room temperature before adding 1 mL of 10% BF3-Methanol. After a flush of nitrogen again, caps were closed, and tubes were placed again in a water bath at 50 °C for 10 min. Tubes were cooled before adding 1 mL of 10% (*w/v*) K_2_CO_3_ and 2 mL of hexane. After centrifugation for 10 min at 1500 rpm and 20 °C, the upper layer containing the fatty acid methyl esters (FAMEs) was collected and stored at −80 °C until injection to GC.

FAMEs were measured on an Agilent 7890A (Santa Clara, CA, USA) equipped with a FID, a programmed temperature injector, and two capillary columns (50 m length, 0.32 mm internal diameter, and 0.25 μm film thickness) coated with 70% cyanopropyl polysilphenylenesiloxane (BPX-70, SGE, Ringwood, VIC, Australia) connected in series. Analysis conditions were as follows: initial temperature of injection (40 °C) was maintained for 0.2 min, then increased up to 200 °C at a rate of 200 °C/min, remained constant at 200 °C for 6 min, before decreasing to 40 °C at a rate of 200 °C/min. Detector temperature was 250 °C. Carrier gas was hydrogen at a pressure of 138 kPa. Oven temperature was programmed as follows: 60 °C for 3 min followed by an increase to 175 °C at a rate of 20 °C/min; the oven was maintained at this temperature for 15 min. Then, temperature was increased up to 215 °C at a rate of 4 °C/min and remained constant for 25 min. Total analysis time was 58.75 min. FAMEs were identified by comparing retention times of peaks with those of pure standards. Quantification of FAMEs was performed by means of calibration curves (peak area vs. concentration), constructed using at least five different concentrations of the aforementioned commercial standards. DHA in the samples was initially expressed as mg/mL of solution. It was then converted into % of recovery for the ready-to-eat matrices, mg/mL of effluents for the duodenal samples, and µg/mL of plasma for the blood samples.

Each sample of duodenal effluents and plasma was trans-methylated in duplicate. DHA quantification by gas chromatography was performed once on each trans-methylated sample.

#### 2.5.3. Protein Concentration and Proteolysis in Effluents

Total protein concentration in the effluents was determined once by the Kjeldahl method (ISO 8968-1:2014, IDF 20-1:2014). Results were expressed as mg protein/100 g effluent.

The rate of proteolysis was assessed by measuring the free primary amino groups in the soluble fraction of the effluents using the orthophthalaldehyde (OPA) method adapted from Church et al. (1983) [52]. This method is based on the reaction of OPA and β-mercaptoethanol with primary amines resulting in 1-alkylthio-2-alkylisondole detected at 340 nm. Briefly, effluents were centrifuged at 10,000× *g* for 10 min and both the pellet and supernatant weights were recorded. Supernatants (one per effluent) were then diluted 1/25, 1/50, and 1/100 in MiliQ water. The OPA assays were carried out in 96-well microplates by adding 50 µL of the diluted samples to 100 µL of OPA reagent (2.5 mL of 20% *w/v* SDS, 50 µL of β-mercaptoethanol, 2.5 mL of OPA at 10 mg/mL in ethanol, and 0.02 M potassium tetraborate buffer pH 9.5 to a final volume of 100 mL). The absorbance was measured at 340 nm after exactly 2 min incubation at room temperature in a Multiskan™ GO Microplate Spectrophotometer (Thermo Fischer Scientific, Waltham, MA, USA). Blanks were made of MiliQ water. Free primary amines were quantified using a calibration curve based on methionine standard solutions (0 to 2.0 mM). Results were expressed as mmol free NH_2_/100 g effluent.

Total protein concentration was converted to mmol of total NH_2_/100 g effluent assuming that the average molecular weight of amino acid residue is 120 g/mol and that the stoichiometry between amino acid and NH_2_ is 1:1. Then a theoretical protein degree of hydrolysis (DH) was calculated as follows:

protein DH = 100 × [(mmol free NH_2_/100 g effluent)⁄(mmol total NH_2_/100 g effluent)]. (1)

### 2.6. Statistical Analyses

All measurements were described as mean values ± SEM. DHA recovery in the matrices was performed in triplicate. All the measures performed in the duodenal effluents used nine pigs (*n* = 9). Plasmatic DHA was measured in seven pigs (*n* = 7) due to blood sampling problems in two pigs.

Time, matrix, and “time × matrix” effects on pH, protein concentration, protein hydrolysis, and base-line adjusted DHA concentration in the duodenal effluents, and on base-line adjusted DHA concentration in plasma were tested using a nonparametric analysis of repeated measures with the “f1.ld.f1” function of the package “nparLD” [53] in R 3.1.2 [54]. In case of a significant effect, the function “npar.t.test” of the package “nparcomp” [55] was used for each time point. Finally, the area under the curve (trapezoidal method) for each matrix was analyzed by a Friedman test. In case of a significant difference, a post hoc Conover test (α-value corrected according to Bonferroni) was applied to perform the multiple comparisons.

## 3. Results

### 3.1. DHA Recovery in the Matrices

In order to determine if DHA was lost during the preparation of the egg matrices, especially in those submitted to a heat treatment, and make sure that all matrices provided the same amount of DHA, the quantity of DHA was measured in the fat fraction extracted from each matrix. Table 2 shows that the DHA recovery was not significantly (*p* > 0.05) different between the three egg matrices, and was close to 100%, which indicates that no loss of DHA occurred. In addition, the absence of any trace of DHA was checked in the foods that the pigs ate on a daily basis after and between assays.

### 3.2. De Visu Morphological Characterization of the Duodenal Effluents

**Figure 1:**
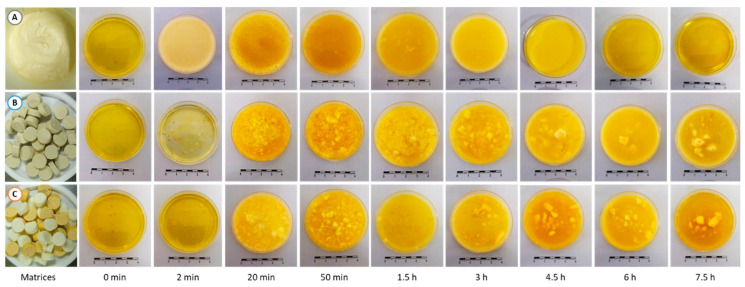
Macroscopic appearance of the three egg matrices and of the corresponding duodenal effluents over a 7.5 h-period of digestion after the ingestion of DHA-enriched matrices: (**A**) mousse; (**B**) omelet; (**C**) hard-boiled egg.

The macroscopic aspect of the duodenal effluents after the ingestion of the DHA-enriched matrices is shown in Figure 1. The samples collected at 15 min before the ingestion of the egg matrices were indicated as 0 min and represented the basal conditions.

As expected, the mousse has led to very homogeneous effluents (Figure 1A), while those obtained after the ingestion of the omelet (Figure 1B) and hard-boiled egg (Figure 1C) were very heterogeneous. In the latter, solid particles were observed in the effluents from 20 min postprandial and up until 7.5 h. Some of these particles were as big as 5–7 mm.

The observation of the duodenal effluents suggested a much faster gastric emptying with the mousse in comparison with the omelet and the hard-boiled egg. Indeed, the duodenal effluent obviously contained the mousse after only 2 min after ingestion, whereas at this digestion time the effluents were identical to that of the basal effluents (0 min) when pigs were fed with the solid egg matrices. In a similar way, the last time point effluent (7.5 h) was very similar to the basal effluent when the pigs were fed with the mousse, whereas large particles were still present in the duodenal effluents after ingestion of hard-boiled egg and omelet.

### 3.3. Physicochemical Evolution of the Duodenal Effluents throughout the Postprandial Period

#### 3.3.1. pH Evolution

**Figure 2:**
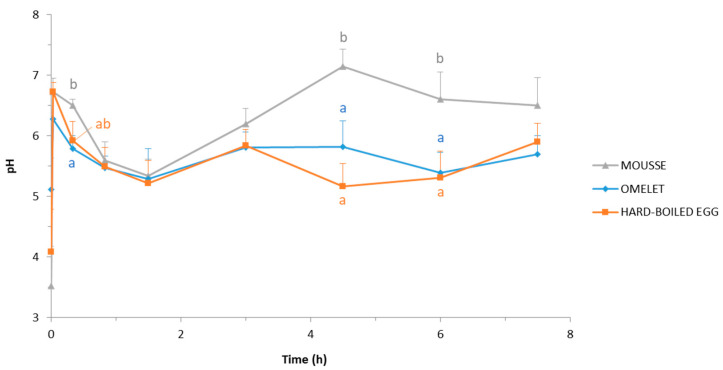
pH evolution of the duodenal effluents throughout 7.5 h postprandial after the ingestion of the DHA-enriched omelet, hard-boiled egg, and mousse. Means and SEM were calculated from *n* = 9 pigs. For a given digestion time, different letters display significantly different mean values (*p* < 0.05).

The mean basal pH values of the duodenal effluents, i.e., measured 15 min before feeding the pigs and here registered as 0 min, were pH 3.5, 4.1, and 5.1 for the experiments with the mousse, the hard-boiled egg, and the omelet, respectively (Figure 2). The ingestion of the matrices resulted in a sudden pH increase until reaching a value around pH 6.5 (pH 6.7, 6.7, and 6.3 for the mousse, the hard-boiled egg, and the omelet, respectively) only 2 min after the beginning of the pig feeding. Except for the mousse, the pH measured at 2 min digestion was the highest pH value observed throughout the postprandial period.

During the first 1.5 h of digestion, the duodenal pH dropped constantly regardless of the egg matrix, even if the decrease was less pronounced during the 20 first min after ingestion of the mousse (Figure 2). At the end of this period, the pH of the duodenal effluents was finally similar for the three egg matrices: 5.3 ± 0.3, 5.3 ± 0.5, and 5.2 ± 0.4 for the mousse, the omelet, and the hard-boiled egg, respectively. These pH values were the lowest ones measured in the duodenum throughout the postprandial period, and regardless of the ingested egg matrix.

From 1.5 h of digestion, duodenal pH increased for the three egg matrices, but two separate behaviors occurred (Figure 2). In the pigs fed with the mousse, the duodenal pH increased more and longer than in pigs fed with the omelet or the hard-boiled egg, to finally reach the highest value (pH 7.1 ± 0.3) after 4.5 h digestion, and before slowly decreasing until the last point recorded (pH 6.5 ± 0.5 after 7.5 h digestion). In the pigs fed with the hard-boiled egg and the omelet, duodenal pH values fluctuated between pH 5.9 and pH 5.2 from 3 h digestion to the end of the sampling, at 7.5 h digestion.

Thus, regarding the duodenal pH, no significant (*p* > 0.05) difference occurred between the omelet and the hard-boiled egg at any digestion time, whereas the mousse resulted in significantly (*p* < 0.05) higher pH values at 4.5 h and 6 h digestion compared to the hard-boiled egg and the omelet, and after 20 min compared to the omelet. After 7.5 h digestion, the duodenal pH values did no longer differ regardless of the egg matrix ingested: pH 5.9 ± 0.3, 5.7 ± 0.3, and 6.5 ± 0.5 when pigs were fed with hard-boiled egg, omelet, and mousse, respectively.

#### 3.3.2. Evolution of Protein Concentration

**Figure 3:**
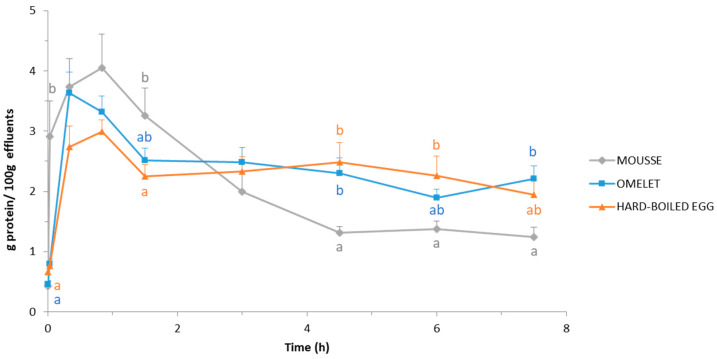
Protein concentration in the duodenal effluents throughout 7.5 h postprandial after the ingestion of the DHA-enriched omelet, hard-boiled egg, and mousse. Concentration is expressed as g protein/100 g effluent. Means and SEM were calculated from *n* = 9 pigs. For a given digestion time, different letters display significantly different mean values (*p* < 0.05).

The evolution of the protein concentration in the duodenal effluents throughout the postprandial period is presented in Figure 3. It may be noted that the basal protein concentration was not zero, but around 0.4–0.6 g/100 g. These values perfectly matches to the concentration of protein previously measured on pig’s pancreatic juice [56,57,58].

The curves highlight that proteins appeared in the duodenum much faster when pigs were fed with the mousse, in comparison to pigs fed the omelet or the hard-boiled egg. When pigs ate mousse, a high protein concentration was detected in the duodenal effluent only 2 min after ingestion: 2.9 ± 0.6 mg/mL vs. 0.8 ± 0.1 mg/mL in pigs fed with omelet and hard-boiled egg. Moreover, after reaching the maximum concentration (at 50 min digestion for the mousse and the hard-boiled egg vs. 20 min for the omelet), the drop was also faster and stronger in the pigs fed with the mousse. Consequently, from 4.5 h digestion to the end of the sampling period (7.5 h digestion), the duodenal concentration of protein was, in most of the cases, significantly (*p* < 0.05) lower in pigs fed with the mousse in comparison to pigs fed omelet or hard-boiled egg. Omelet and hard-boiled egg led to similar (*p* > 0.05) protein concentration in the duodenum throughout the entire postprandial period.

#### 3.3.3. Evolution of Proteolysis

**Figure 4:**
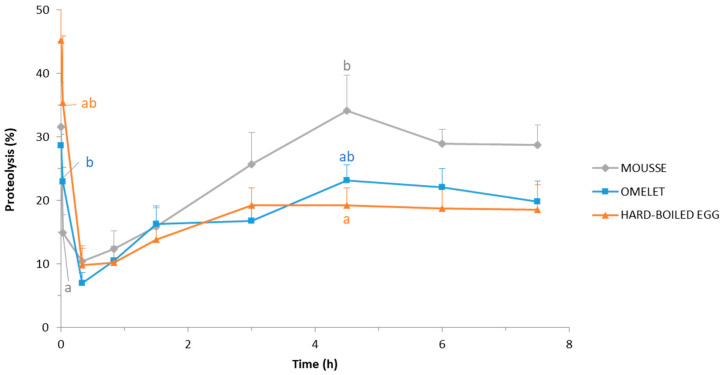
Degree of proteolysis (%) in the duodenal effluents throughout 7.5 h postprandial after the ingestion of the DHA-enriched omelet, hard-boiled egg, and mousse. Means and SEM were calculated from *n* = 9 pigs. For a given digestion time, different letters display significantly different mean values (*p* < 0.05).

The evolution of the degree of proteolysis in the duodenal effluents throughout the postprandial period is presented in Figure 4. Except the 0 min and 2 min time-points to which residual endogenous proteins were most likely predominant, the degree of proteolysis was similar (*p* > 0.05) for the three egg matrices during the first 3 h of digestion. Within that period, it increased from about 10% at 20 min digestion (7.0 ± 1.7, 9.8 ± 2.7, and 10.4 ± 2.5% for omelet, hard-boiled egg, and mousse, respectively) to about 15–25% after 3 h digestion (16.8 ± 2.3, 19.3 ± 2.7, and 25.7 ± 5.0% for omelet, hard-boiled egg, and mousse, respectively).

From that time on, the degree of proteolysis continued to increase up to 4.5 h for the mousse and the omelet but remained almost constant for the hard-boiled egg (Figure 4). The highest degree of proteolysis was measured at 4.5 h of digestion for the mousse and the omelet. At this point, protein hydrolysis was significantly (*p* < 0.05) higher in the pigs fed mousse (34.1 ± 5.6%) than in the pigs fed hard-boiled egg (23.1 ± 2.5); the omelet led to an intermediate value (19.3 ± 2.7%).

#### 3.3.4. Evolution of DHA Concentration

**Figure 5:**
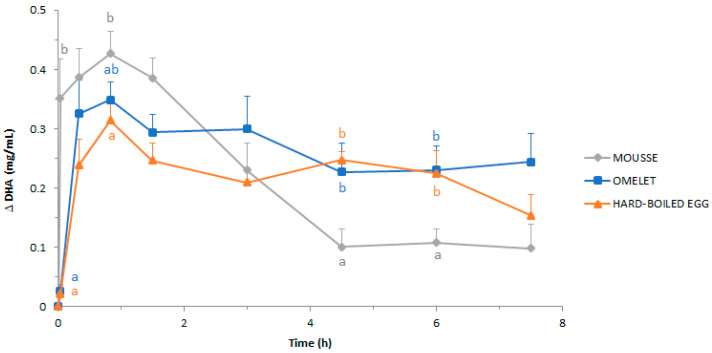
Base-line adjusted DHA concentration in the duodenal effluents throughout 7.5 h postprandial after the ingestion of the DHA-enriched omelet, hard-boiled egg, and mousse. Concentration is expressed as mg DHA/mL effluent. Means and SEM were calculated from *n* = 9 pigs. For a given digestion time, different letters display significantly different mean values (*p* < 0.05).

The evolution of the base-line adjusted DHA concentration in the duodenal effluents (thereafter refered to as duodenal DHA concentration) during digestion, expressed as mg/mL of effluent, is plotted in Figure 5. It should be noted that the DHA concentration measured in the duodenal effluents cannot be considered as a measurement of bioaccessibility, since total DHA, and not only soluble DHA, was measured.

The similarity of the kinetics of duodenal DHA concentration (Figure 5) with those of protein concentration reported above (Figure 3) is striking. Briefly, DHA appeared in the duodenum much faster when it was included in the mousse, in comparison to the omelet and the hard-boiled egg. In pigs fed with the mousse, a high concentration of DHA was detected in the duodenal effluent only 2 min after ingestion (0.35 ± 0.07 mg/mL), whereas at the same time only traces were detected in pigs fed with omelet and hard-boiled egg (0.03 ± 0.01 and 0.02 ± 0.01 mg/mL, respectively). Then, the duodenal concentration of DHA continued to rise until reaching maximum values at 50 min digestion regardless of the matrix. The maximum values were 0.43 ± 0.04, 0.35 ± 0.03, and 0.31 ± 0.04 mg/mL for the mousse, the omelet, and the hard-boiled egg, respectively, i.e., significantly (*p* < 0.05) higher in pigs fed with the mousse, in comparison to the pigs fed with the hard-boiled egg.

From 50 min digestion onwards, the duodenal concentration of DHA decreased for the three egg matrices, but the fall was much more marked and lasted longer in pigs fed with the mousse than in pigs fed with omelet or hard-boiled egg (Figure 5). Consequently, at 4.5 h digestion, the mousse resulted in the lowest duodenal concentration of DHA at 0.1 mg/mL, i.e., a drop of about 75% with respect to the maximum concentration (at 50 min digestion). Then, the DHA concentration remained stable until 7.5 h digestion.

In pigs fed with the omelet, the duodenal concentration of DHA decreased less steeply until 4.5 h digestion, before remaining stable up to the last sampling (Figure 5). Then, after 7.5 h digestion, the duodenal concentration of DHA had fallen only 30%, to reach 0.24 ± 0.05 mg/mL that is around a DHA concentration 2.5-fold higher than that measured at the same digestion time in pigs fed with the mousse. However, this difference was not statistically significant.

When pigs were fed the hard-boiled egg, the duodenal concentration of DHA decreased in a way that, at 3 h digestion, it reached the same level as that of pigs fed with the mousse (Figure 5). Then it remained almost stable until 6 h digestion, before decreasing again. At the end of the recorded time (7.5 h), the duodenal concentration of DHA had fallen 51.2% to reach a value that was not significantly (*p* > 0.05) different from that obtained in pigs fed with the mousse and the omelet.

### 3.4. Evolution of the Plasma Concentration of DHA throughout the Postprandial Period

**Figure 6:**
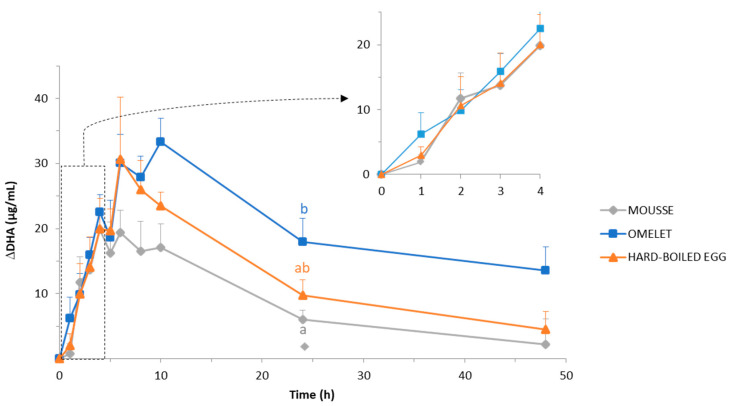
Base-line adjusted DHA concentration in plasma, expressed in µg DHA/mL plasma, throughout 48 h postprandial after the ingestion of the DHA-enriched omelet, hard-boiled egg, and mousse. Time points between 0 and 4 h are zoomed in the upper right-side of the figure. Means and SEM were calculated from *n* = 7 pigs. Time effect was significant (*p* < 0.001; nparLD analysis and post hoc Tukey test). Time × matrix and matrix effects were not significant. For a given digestion time, different letters display significantly different mean values (*p* < 0.05).

The evolution of the base-line adjusted DHA concentration in plasma (thereafter refered to as DHA concentration in plasma) after the ingestion of the three DHA-enriched egg matrices is presented in Figure 6. The three curves followed a similar pattern, i.e., first a sharp increase before progressively decreasing up to 48 h after the meal. At this end-time point, except in pigs fed with the mousse, DHA concentration did not yet return to the basal level (*p* < 0.05).

The highest DHA concentration (33.3 ± 3.6 µg/mL) was measured in pigs fed with the omelet, at 10 h digestion. The maximum value in pigs fed with the hard-boiled egg (30.6 ± 9.6 µg/mL) was not significantly (*p* > 0.05) different, but it was observed earlier (6 h) than in pigs fed with the omelet. In contrast, the maximum DHA concentration was significantly (*p* < 0.05) lower in pigs fed with the mousse (19.8 ± 2.7 µg/mL) than in pigs fed omelet or hard-boiled egg. Moreover, it was measured even earlier for the mousse, at 4 h digestion. Since the DHA elimination rates from the maximum values were very similar regardless of the matrix, the differences in DHA plasma concentration among the three matrices remained very similar until the end of the sampling period. The highest differences among the three matrices were reached 24 h after feeding. At this point, DHA plasmatic concentration in pigs fed with the omelet was significantly higher (*p* < 0.05) than in those fed with the mousse (17.9 ± 3.7 µg/mL and 6.1 ± 1.4 µg/mL, respectively). At the last sampling point (48 h), DHA plasmatic level in pigs fed with the omelet (14.1 ± 3.2 µg/mL) tended to be higher than in pigs fed hard-boiled egg and mousse (6.0 ± 1.5 µg/mL and 4.6 ± 3.2 µg/mL, respectively), despite no significant difference (*p* > 0.05).

In view of the overall kinetics of the DHA plasma concentration, the “time × matrix” interaction was not significant (*p* = 0.34). However, the comparison of the areas under the curves (AUC; Table 3) calculated according to the trapezoidal method, and using the Friedman test and the Conover post-hoc test, highlighted that a significantly higher AUC value was measured in pigs fed with omelet in comparison with pigs fed hard-boiled egg or mousse (*p* < 0.016). On the contrary, the difference between pigs fed with hard-boiled egg and those fed with mousse was not significant (*p* = 0.46).

The differences in the AUC resulted primarily from the fact that, after ingestion of the omelet, the accumulation of DHA in plasma started earlier and continued over a longer period than after ingestion of the mousse or hard-boiled egg (Figure 6). Tukey’s post hoc tests showed that after 1 h of digestion, only the omelet had produced a significant (*p* < 0.05) increase of DHA plasma concentration with respect to the initial basal level. With mousse and hard-boiled egg, the increase was not significant (*p* > 0.05) until 2 h and 3 h digestion, respectively. In addition, the accumulation of DHA in plasma after meal ingestion only lasted 4 h and 6 h when pigs were fed with mousse and hard-boiled egg, respectively, vs. 10 h when pigs were fed omelet, i.e., for a period 2.5-fold and 1.7-fold longer than with the mouse and the hard-boiled egg, respectively (Figure 6).

Assuming that the AUC of DHA plasma concentration was a measurement of DHA bioavailability, it can be concluded that DHA bioavailability was significantly higher when DHA was included in the omelet in comparison with the hard-boiled egg (+56%; *p* < 0.05), and in the omelet in comparison with the mousse (+120%; *p* < 0.001) (Table 3).

## 4. Discussion

The main objective of the present study was to investigate the effect of the food matrix on the DHA bioavailability. In that aim, three DHA-enriched egg matrices with the same composition but different structures were designed. Omelet and hard-boiled egg were solid matrices in which heat gelation of egg white proteins occurred in the presence or in the absence of lipids, respectively. Mousse was a liquid foamy matrix, without any cooking.

Due to the ethical constraints related to clinical studies, and despite the issues of the present study largely target human nutrition and health [21], it was decided to use a pig model for an in vivo study. In vivo models are the gold standard to measure the nutrient bioavailability [59,60]. Indeed, no available in vitro model considers the complexity of the mechanisms that occur between the ingestion of nutrients and their appearance in the bloodstream. The downside of the in vivo models is that indigestible markers, such as chromium oxide or titanium oxide, need usually to be added to the test diet in order to measure some physiological parameters such as the rate of gastric emptying and the volume of endogenous gastric and intestinal secretions. These controls are required for accurate material balance and measurements [61]. However, in the present study, it was decided not to add such indigestible markers since most of metals act as promoters of lipid oxidation [62,63].

Pigs were fed with the three DHA-enriched egg matrices and DHA bioavailability was estimated from the DHA concentration in plasma over a period of 48 h postprandial. Additionally, in order to better understand the potentially different behaviors of the three egg matrices during digestion, and then to explain the expected differences regarding DHA bioavailability, the duodenal effluents were analyzed. First, a morphological de visu characterization of the effluents was carried out. Then, pH, protein concentration, and DHA concentration in the effluents were measured. These parameters could be regarded as indicators of gastric emptying. Lastly the degree of proteolysis was estimated. This parameter could be regarded as indicative of the extent of food digestion.

One of the most flagrant results was the much faster transit of the mousse from the stomach to the duodenum in comparison with the omelet and the hard-boiled egg. This is proven by visual observation of the duodenal effluents, which contained mousse as early as 2 min after feeding started, while the pigs were still eating. On the contrary, the duodenal effluents did not appear to contain egg at this early digestion time when pigs were fed omelet or hard-boiled egg (Figure 1). These observations were supported by measurements in the effluents: proteins as well as DHA were detected only 2 min after feeding started in the case of the mousse, unlike omelet and hard-boiled egg (Figure 3 and Figure 5). This rapid and massive arrival of the mousse into the duodenum from the very beginning of digestion might also explain the higher duodenal pH observed at 20 min digestion in pigs fed the mousse (Figure 2). Because of a very fast transit in the stomach, the first eluted fractions of mousse would not be acidified, resulting in a duodenal pH equivalent to the mousse pH in the first moments of digestion. Indeed, in vivo experiments in pigs fed with egg white gels showed that 20 min after ingestion, the intra-gastric pH was almost homogeneous and similar to the pH of the meal [64].

It should be noted that the mousse, which had a very soft and fragile texture, was apparently destabilized into a liquid when reaching the duodenum, likely due to the salivary and gastric juices. On the contrary, gel pieces were still visible in the duodenal effluents after omelet and hard-boiled egg meals (Figure 1). Then, the results were in line with those of Barbé et al. [65], Hellström et al. [66], and Kong and Singh [67] who reported difference in behavior between liquid and solid foods. Barbé et al. [65], who used EDTA-Cr as indigestible marker and pig as digestion model, observed that the mean retention time in the stomach of a gelled dairy matrix was 51 min longer than that of a liquid dairy matrix, both with the same composition. According to Hellström et al. [66] and Kong and Singh [67], liquid and semi-liquid matrices empty from the stomach according to first-order kinetics at a speed directly proportional to the food volume present in the stomach. Immediately after ingestion, liquid matrices would empty at rates of up to 10 to 40 mL/min before, after a while, slowing down to rates around 2 to 4 mL/min. On the contrary, gastric emptying of solid foods usually show a typical biphasic pattern: after a lag phase during which little emptying occurs, a linear emptying phase follows during which solid particles empty from the stomach by mainly zero-order kinetics, i.e., at a speed independent of the gastric volume.

Actually, during the first 1.5 h digestion of the mousse, the duodenal pH decreased to around 5.2, likely due to insufficient duodenal secretions to buffer the acidic pH of the gastric chyme that emptied. From that time on, a clear inverse relationship was observed between the duodenal pH on one hand (Figure 2), and the protein and DHA concentrations on the other (Figure 3 and Figure 5). Thus, from 4.5 h digestion, the duodenal pH was around 6.5–7.0 when pigs were fed the mousse, suggesting that only small quantities of gastric chyme emptied from the stomach during this period. This was supported by the protein and DHA concentrations in the duodenum that were minimal during that period, very close to the basal levels. In other words, gastric emptying was almost complete after 4.5 h digestion when pigs were fed the mousse, consistently with the aspect of the duodenal effluents (Figure 1). On the contrary, the more or less constant pH values around 5.5–6.0 until 7.5 h digestion when pigs were fed omelet or hard-boiled egg (Figure 2) suggest that still large amounts of the solid egg matrices were continuously released from the stomach up to 7.5 h after feeding. The almost stable and much higher concentrations of proteins and DHA that reached the duodenum during that period for omelet and hard-boiled egg meals (Figure 3 and Figure 5) support this assumption of a second linear phase of gastric emptying, typical of solid foods according to Hellström et al. [66] and Kong and Singh [67]. However, contrary to these authors who stated that all solids are removed from the stomach after approximately 3 to 4 h digestion, our results showed that still large pieces of omelet and hard-boiled egg were visible in the duodenum after 7.5 h digestion (Figure 1). Since it seems unlikely that food particles of such a big size (5–7 mm) could have been trapped in the duodenum for a long time because of the peristaltic movements, these observations indicate that gastric emptying of solid food particles might be much longer than reported in the literature.

The mousse could also be distinguished from the omelet and the hard-boiled egg in terms of proteolysis kinetics (Figure 4). From 3 h up to 7.5 h digestion, the proteolysis degree measured in the duodenum was higher in pigs fed the soft and fragile mousse, in comparison to pigs fed both solid egg matrices. One can assume that the proteolytic digestive enzymes should have an easy and instant access to proteins in a liquid food such as the mousse once mixed with salivary and gastric juices, whereas proteolysis would be limited to the surface of food particles in the case of the solid egg matrices. Indeed, the food matrix is known to affect the diffusion of digestive enzymes. Especially, pepsin diffusion has been shown higher in liquid as compared to semi-solid gels, and protein gels with a smaller pore size, due to either a higher protein fraction or particular physicochemical conditions during gelation, have a greater hindrance on pepsin mobility, decreasing the enzyme diffusion [68,69,70]. Actually, pepsin would have limited penetration depth into food gels for which gastric digestion would be mainly limited to surface erosion [71].

Finally, the most striking result, in line with the main issue of the present study, was that the food matrix actually had a significant impact on the DHA bioavailability (Table 3). Thus, DHA bioavailability was 57% higher when DHA was provided through DHA-enriched omelet than through DHA-enriched hard-boiled egg, and 120% higher than through DHA-enriched mousse. On the contrary, hard-boiled egg and mousse did not significantly differ.

The poor bioavailability of DHA included in the mousse might be a consequence of the fast gastric and duodenal emptying of this soft and fragile egg matrix. The rapid and massive arrival of the DHA-enriched mousse in the duodenum (Figure 5) may have resulted in an incomplete release of DHA from PLs, because of an insufficient secretion of pancreatic phospholipase in the very first moment of the digestion. Then, while the secretion of pancreatic juice progressively increased, the amounts of duodenal DHA sharply dropped (Figure 5) because of the rapid emptying of the mousse out of the duodenum (Figure 3). Consequently, DHA incorporation into the mixed micelles would be lower in pigs fed mousse, as compared to pigs fed omelet. Then, the solubilization of DHA into mixed micelles is absolutely needed for DHA uptake through the endothelial epithelium of the intestine [72]. Additionally, the saturation of the transport system into enterocytes could be another explanation. Indeed, the uptake of short fatty acids (up to eight carbon atoms) by the enterocytes linearly depends on the luminal concentration [73]. On the contrary, the uptake/binding of long-chain fatty acids at the brush border membrane is a mechanism which may be saturated [74,75,76]. Actually, it is striking that the patterns of DHA accumulation in plasma were very similar regardless of the egg matrix (Figure 6) despite this not being the case for DHA concentration in the duodenum (Figure 5). Especially, the amount of DHA for absorption was likely much higher for the mousse than for the other egg matrices during the first 1.5 h of digestion, whereas the mousse resulted in the lowest DHA plasma concentration during this early phase of digestion. Overall, the discrepancy between the appearance of DHA in the duodenum on one hand, and in the plasma (DHA bioavailability) on the other hand suggests that the “intestinal absorption system” was saturated when pigs were fed with the rapidly emptied mousse, whether digestive enzymes be released in insufficient quantities or the absorption by the intestinal wall be overflowed. Alternatively, not DHA absorption, but cellular export of DHA from enterocytes could have been exceeded. Following digestion, dietary lipids are taken up by the enterocytes that soon begin to produce the triglyceride-rich chylomicron (CM) particles. CM production within the enterocytes is a highly regulated process, the major driver of which is the amount and type of ingested lipids [77]. One can assume that the mousse led to a massive and rapid release of DHA from the matrix, and consequently led to a fast DHA absorption by the enterocytes, but without being coupled to a sufficient CM synthesis for an efficient export of DHA into the blood stream.

The difference between omelet and hard-boiled egg regarding the DHA bioavailability was smaller than that between omelet and mousse, but it was significant (Table 3). However, the digestion kinetics of both gelled egg matrices (omelet and hard-boiled egg) did not seem to significantly differ, based on the evolution of duodenal pH (Figure 2), protein concentration (Figure 3) and proteolysis (Figure 4) throughout the postprandial period. All these parameters suggested similar emptying patterns from the stomach to the duodenum and out of the duodenum, as well as similar kinetics of food disintegration. An assumption could be that DHA was more efficiently protected against degradation in the acidic environment of the stomach when included in the omelet in comparison with hard-boiled egg, resulting in a DHA release in the duodenum that lasted longer with the omelet meal. Indeed, the stomach environment has been proved conducive to lipid oxidation. Moreover, the uptake of oxidized DHA by Caco-2 cells has been shown lower than that of non-oxidized DHA, given that Caco-2 cells are regarded as a relevant model for lipid assimilation by intestinal enterocytes [78]. The only difference between the omelet and the hard-boiled egg was that egg white and yolk were mixed before cooking in the case of the omelet, resulting in a quite firm, elastic and homogeneous gel. In contrast, both egg fractions were cooked separately before mixing in the case of the hard-boiled egg, resulting in a mixture of gel pieces made either of firm and elastic egg white gel, or soft and brittle yolk gel, the DHA being included in the latter. One assumption could then be that the mixed gel of the omelet might be more protective for DHA than the yolk gel of the hard-boiled egg.

## 5. Conclusions

For the first time to the best of our knowledge, the present study established the impact of food matrix on DHA bioavailability, using three matrices of same composition but different structure. It should be noted that, with the food models we used, i.e., three realistic DHA-enriched egg products, the differences were enormous: omelet resulted in a DHA bioavailability 120% higher than that of the less efficient product, i.e., the mousse. These results may have a major impact on nutritional strategy when DHA supplementation is aimed. Indeed, it suggests that the choice of the vector food should be carefully made in order to maximize the efficiency of DHA absorption by the body, at least when DHA is provided under the form of phospholipids. In this case, liquid foods do not seem the best choice, likely because of a too fast gastrointestinal transit. Indeed, it seems that the higher the residence time of the lipids in duodenum, the higher DHA bioavailability. However, it would be relevant to check whether same conclusions could be drawn for the long term. Indeed, in the present study, the pigs ate each of the three egg matrices only once. Then, DHA supplementation usually runs over several weeks when the purpose is a nutritional intervention. Similarly, the question arises if the impact of food matrix would be of the same magnitude with “normal” foods, i.e., not DHA-enriched foods. Lastly, the (supposed) superiority of DHA-enriched omelet warrants further confirmation regarding the health benefits of DHA. The use of genetically modified pig models for hypercholesterolemia and atherosclerosis [79], T2DM [80], or over-expression of endothelial nitric oxide synthase [81] could be useful in that aim, before clinical studies.

## Figures and Tables

**Table 1:** OVO-DHA^®^ fatty acid composition. Source: ASL (Vichy, France).

Common Name	Chemical Name	Formula	mg/g of Product	Relative % Total Fatty Acids (*w/w*)
Myristic acid	Tetradecanoic acid	C 14:0	2.29	0.41
Valeric acid	Pentanoic acid	C 15:0	0.83	0.15
Palmitic acid	Hexadecanoic acid	C 16:0	148.72	26.85
Palmitoleic acid	9-*cis*-Hexadecenoic acid	C 16:1	22.33	4.03
Margaric acid	Heptadecanoic acid	C 17:0	0.62	0.11
Stearic acid	Octadecanoic acid	C 18:0	38.14	6.89
Oleic acid	*cis*-9-Octadecenoic acid	C 18:1	243.09	43.89
Linoleic acid	*all-cis*-9,12-octadecadienoic acid	C 18:2 (n-6)	52.23	9.43
ɣ-linolenic acid, GLA	*all-cis*-6,9,12-octadecatrienoic acid	C 18:3 (n-6)	0.32	0.06
α-linolenic acid, ALA	*all-cis*-9,12,15-octadecatrienoic acid	C 18:3 (n-3)	2.45	0.44
Eicosenoic acid	*cis*-11-eicosenoic acid	C 20:1	1.38	0.25
Dihomo-ɣ-linolenic acid, DGLA	*all-cis*-8,11,14-eicosatrienoic acid	C 20:3 (n-6)	0.30	0.05
Arachidonic acid, AA	*all*-*cis*-5,8,11,14-eicosatetraenoic acid	C 20:4 (n-6)	3.46	0.62
Timnodonic acid, EPA	*all-cis*-5,8,11,14,17-eicosapentaenoic acid	C 20:5 (n-3)	1.85	0.33
Clupanodonic acid, DPA	*all-cis*-7,10,13,16,19-docosapentaenoic acid	C 22:5 (n-3)	1.57	0.28
Osbond acid	*all-cis*-4,7,10,13,16-docosapentaenoic acid	C 22:5 (n-6)	0.42	0.08
Cervonic acid, DHA	*all-cis*-4,7,10,13,16,19-docosahexaenoic acid	C 22:6 (n-3)	26.25	4.74
Total mg			553.92	

**Table 2:** Docosahexaenoic acid (DHA) recovery (%) in the three DHA-enriched matrices.

Omelet	Hard-Boiled Egg	Mousse
99.4 ± 2.5	95.13 ± 0.0	91.7 ± 3.6

Means and SEM were calculated from *n* = 3 measurements for omelet and mousse, *n* = 2 measurements for hard-boiled egg.

**Table 3:** Bioavailability of DHA included in DHA-enriched omelet, hard-boiled egg, and mousse, evaluated by measuring the area under the curve (AUC) of base-line adjusted DHA concentration in plasma throughout 48 h postprandial after the ingestion of each egg matrix.

	Omelet	Hard-Boiled Egg	Mousse
AUC (calculated from Figure 6)	950.1 ± 140 ^a^	609.5 ± 78.6 ^b^	432.5 ± 43 ^b^

Means ± SEM were calculated from *n* = 7 pigs. Different letters display significantly different mean values (Conover post-hoc test, *p* < 0.016, Bonferroni corrected).
